# Sequential tests and biologically grounded multi-alternative decision making

**DOI:** 10.1186/1471-2202-12-S1-P137

**Published:** 2011-07-18

**Authors:** Javier A Caballero, Nathan Lepora, Kevin N Gurney

**Affiliations:** 1 Department of Psychology, The University of Sheffield, Sheffield, South Yorkshire, S10 2TN, UK

## 

**Figure 1:**
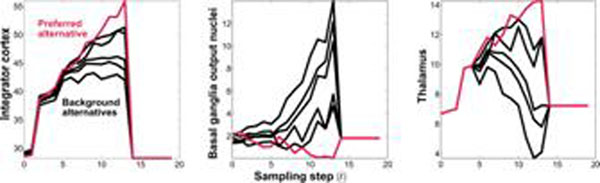
Time course of firing-rate-like signals on selected stages, during a single decision trial up to a threshold, using the closed loop, delayed MSPRT (1 sample every 15 ms). Compare with figure 7 in [4].

Recently, Bogacz and Gurney [1] proposed that the cortico-basal-ganglia system implements asymptotically optimal decision making between several alternatives, based on sensory evidence, through a statistical algorithm known as the multi-hypothesis sequential probability ratio test (MSPRT). The original programme of work focused on architectural features of the system and made simplifying assumptions as to its physiology. Here, we extend that work to include more biologically-realistic properties and pathways, and explain their impact on the MSPRT performance. One assumption in [1] was that ‘noise’ on sensory signals was Gaussian distributed, which poses the problem of interpreting the signals in the negative tail of the distribution in terms of neural events. We addressed this issue in previous work [2] by deducing a new MSPRT that proposed these signals are Inverse Gaussian distributed (wholly positive and skewed). Using realistic parameters we showed this MSPRT requires about a tenth of the samples of its Gaussian predecessor to reach a decision with the same proportion of errors. A further simplification in [1] was that the processing elements were dynamics-free and the inter-element delays were zero. In reality neural membranes display non-trivial dynamics and significant inter-neuronal/synaptic processing delays are present, both having an effect on the performance. Hence we developed a system of first order differential equations with the time constants for membranes at rest available in the literature. We found that such a system had decision sample sizes roughly twice that of the non-dynamic MSPRT. However, active membranes have reduced time constants [3]. Using values one half of those at rest gave a 30% reduction in performance, and values of one fifth of those at rest, only a 4% decrease. Furthermore, following a closer examination of the anatomy, we added new pathways to give the architecture cortex→basal-ganglia→thalamus↔cortex (arrows indicating information flow), which completes the mapping of a ‘Bayesian structure’ onto it and represents the full decision/inference circuit. Closing such loop recursively feeds back the posterior inference from sampling step *t* as prior ‘knowledge’ for further steps *t* + *Δt*. By doing this we also constructed another recursive statistical test (also a MSPRT) that differs from the non-recursive (feedback-less) one proposed in [1]. To test the effect on performance of including these pathways, we incorporated them into a non-dynamic MSPRT with Inverse Gaussian inputs from [2], including the main delays. The recursion increased the number of samples to reach a decision by around 30% compared with its original non-recursive counterpart. This is nevertheless a small sacrifice considering that in return the system becomes more general and ultimately able to bias further decisions by adjusting its prior knowledge. Finally, in figure 1 we show the evolution of several representative elements of the loop during a decision. These results suggest that the closed loop, delayed MSPRT might hold the key to explain the time course and magnitude of the firing rates observed, in macaque electrophysiological single unit recordings, during a decision task [4].
